# Supplementary material for: First steps towards mitochondrial pan-genomics: detailed analysis of Fusarium graminearum mitogenomes
Source: PeerJ. 2018 Dec 19;6:e5963. doi: 10.7717/peerj.5963 (PMC6304159; doi:10.7717/peerj.5963)
Supplement: Text S1 [file peerj-06-5963-s003.pdf]

ASSEMBLY OF THE POOLED DATA

GRAbB (Brankovics et al., 2016) was used with SPAdes 3.8.1 assembler (Bankevich et al., 2012; Nurk et al., 2013) to reconstruct the mitogenome of the strains that were pooled before sequencing. GRAbB was chosen because it is a wrapper program for iterative *de novo* assembly based on a reference sequence. SPAdes assembler was used, since it offers good insight for the user into the relationship between nodes in the assembly graph and the relationship between nodes, contigs and scaffolds. When GRAbB finished the assembly process, the final assembly graph (Fig. 1) was visualized using Bandage (Wick et al., 2015).

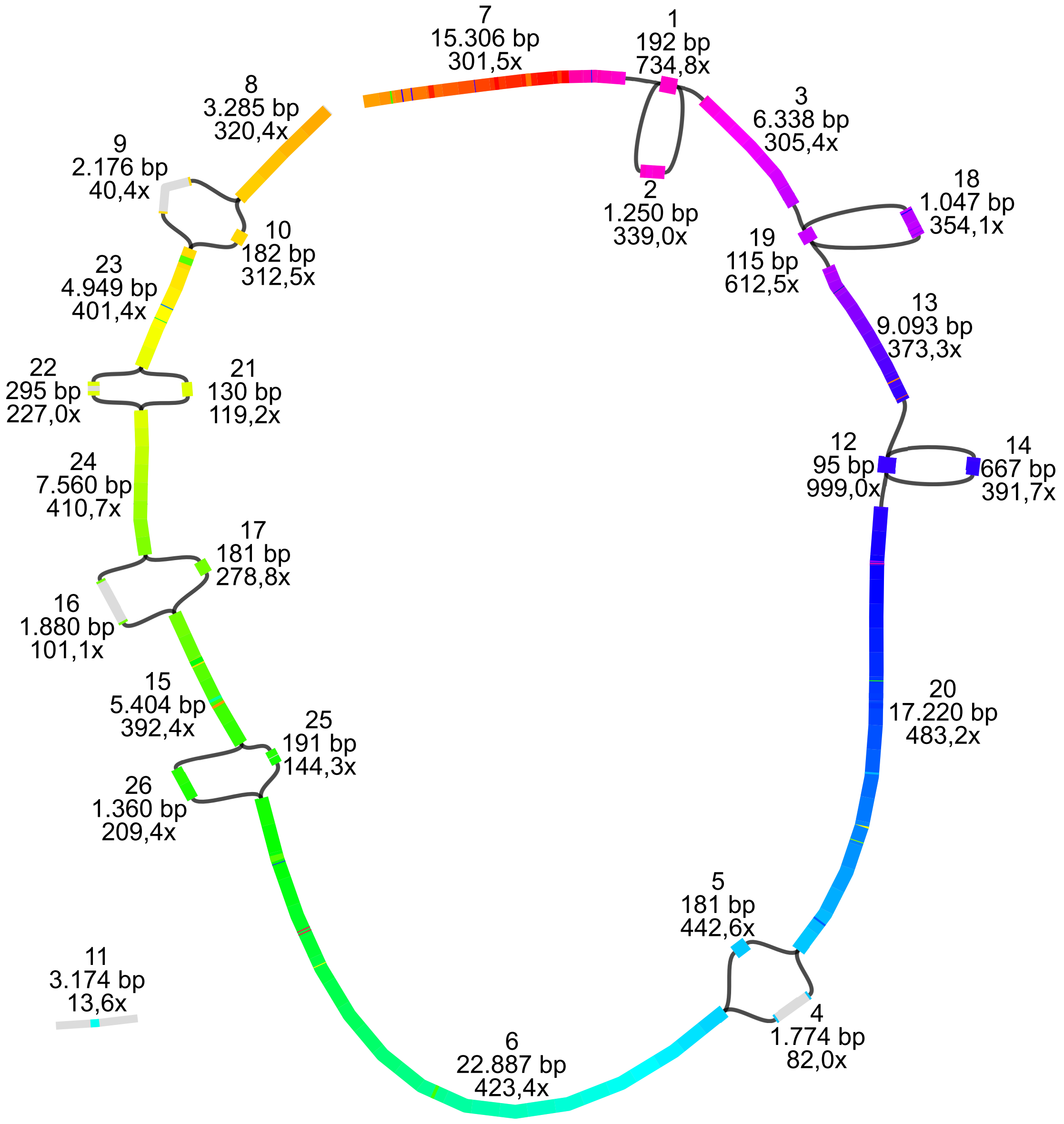

Figure 1. Assembly graph of the pooled dataset. The image was created using Bandage (after running BLAST against the mitogenome of PH-1). The nodes were colored by Bandage using the BLAST hits (rainbow) setting. Node 11 represents a NUMT (nuclear mitochondrial DNA segment).

The loops observed at nodes 1, 12 and 19 were short repeat regions, these were resolved by SPAdes when creating the *contigs.fasta* file. Node 11 represented a nuclear mitochondrial sequence (NUMT). This could be estimated based on its coverage value (13.6x). BLASTN analysis showed that this sequence part of chromosome 1 of the PH-1 genome. Nodes 9 and 10, 16 and 17, 21 and 22, 25 and 26 represent alternative alleles. The sum of the coverage values of the allele pairs is approximately equal to the coverage values of the mono-morphic regions. The polymorphic regions were too far apart to establish

linkage between them, so in order to capture the sequence diversity found in the dataset two alternative assemblies ("short" and "long") were extracted from the graph: one with the shorter allele at all of the positions and one with the longer allele at all of the positions (Supplementary Table 1). Both of the two sequences could be circularized, because the sequences of nodes 7 and 8 showed 185 bp long overlap. The overlapping region contained 3 SNPs that needed to be resolved by read mapping to identify the correct nucleotide for those positions. These were also confirmed when compared to the mitogenome sequence of PH-1 strain. The assembly method did not reveal SNP variations, only intron presence/absence variations and an alternative idiomorph variation.

REFERENCES

Bankevich, A., Nurk, S., Antipov, D., Gurevich, A. A., Dvorkin, M., Kulikov, A. S., Lesin, V. M., Nikolenko, S. I., Pham, S., Prjibelski, A. D., Pyshkin, A. V., Sirotkin, A. V., Vyahhi, N., Tesler, G., Alekseyev, M. A., and Pevzner, P. A. (2012). SPAdes: A new genome assembly algorithm and its applications to single-cell sequencing. *Journal of Computational Biology*, 19(5):455–477.

Brankovics, B., Zhang, H., van Diepeningen, A. D., van der Lee, T. A. J., Waalwijk, C., and de Hoog, G. S. (2016). GRAbB: Selective assembly of genomic regions, a new niche for genomic research. *PLoS Comput Biol.*, 12(6):e1004753.

Nurk, S., Bankevich, A., Antipov, D., Gurevich, A. A., Korobeynikov, A., Lapidus, A., Prjibelski, A. D., Pyshkin, A., Sirotkin, A., Sirotkin, Y., Stepanauskas, R., Clingenpeel, S. R., Woyke, T., McLean, J. S., Lasken, R., Tesler, G., Alekseyev, M. A., and Pevzner, P. A. (2013). Assembling single-cell genomes and mini-metagenomes from chimeric MDA products. *Journal of Computational Biology*, 20(10):714–37.

Wick, R. R., Schultz, M. B., Zobel, J., and Holt, K. E. (2015). Bandage: Interactive visualization of *de novo* genome assemblies. *Bioinformatics*, 31(20):3350–3352.
